# Supplementary figures and images for: Prestige and homophily predict network structure for social learning of medicinal plant knowledge
Source: PLoS One. 2020 Oct 8;15(10):e0239345. doi: 10.1371/journal.pone.0239345 (PMC7544085; doi:10.1371/journal.pone.0239345)

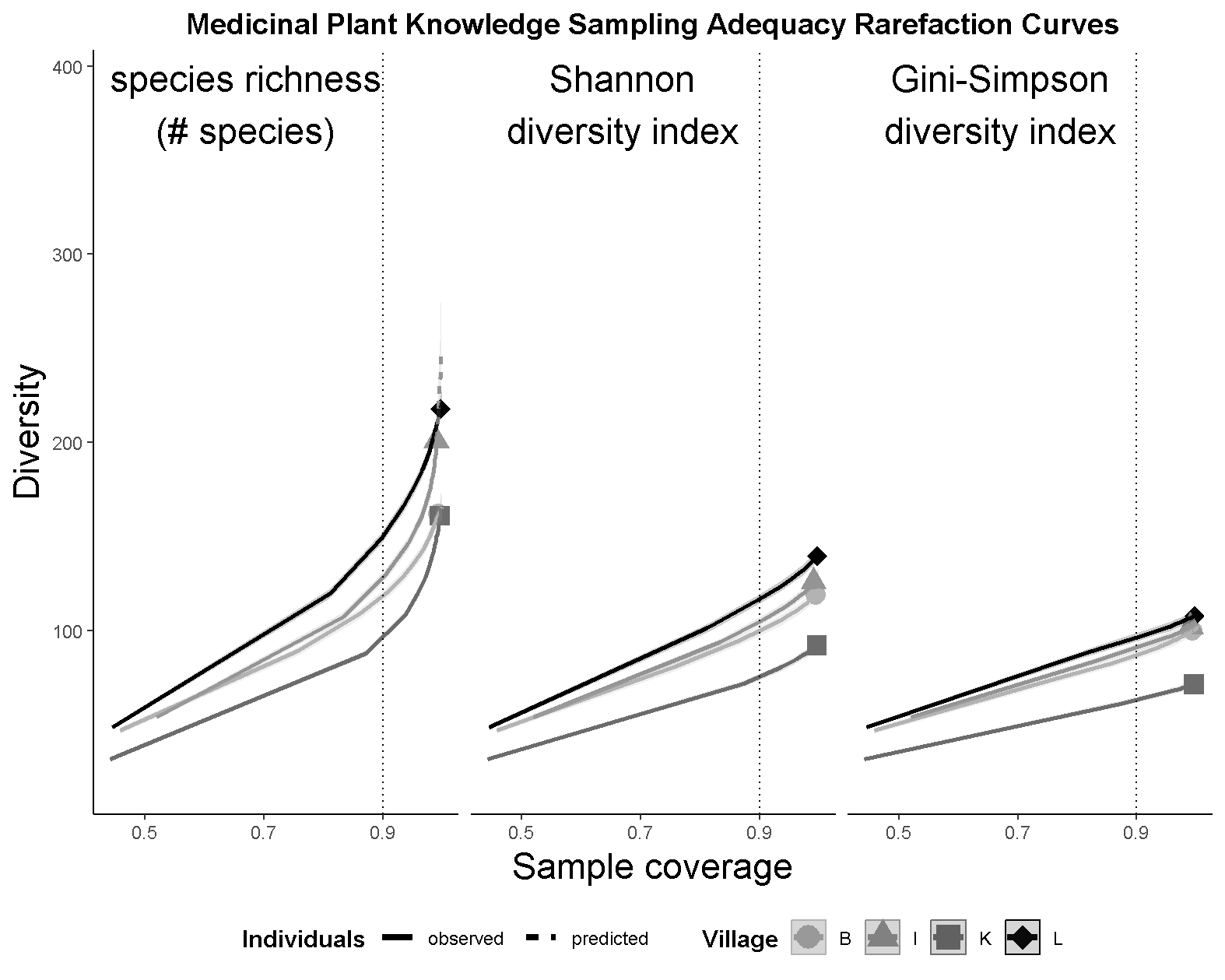

Supplement: S1 Fig — Curves show coverage-based rarefaction (solid lines) and extrapolation (dashed lines). 95% confidence intervals (the shaded area associated with each curve) were estimated using 500 bootstrap replicates. Shannon and Simpson diversity account for both richness and evenness of the species cited. Species richness, Shannon diversity, and Gini-Simpson diversity ranged from161-218, 92.2–139.9, and 71.6–108.0, respectively. Key: Shannon diversity index = exponential of Shannon entropy, Gini-Simpson diversity index = inverse Simpson concentration, Key: B = Binaoli village, I = Irobulu village, K = Kolofi village, L = Lagoe village. (TIF) [file pone.0239345.s001.tif]

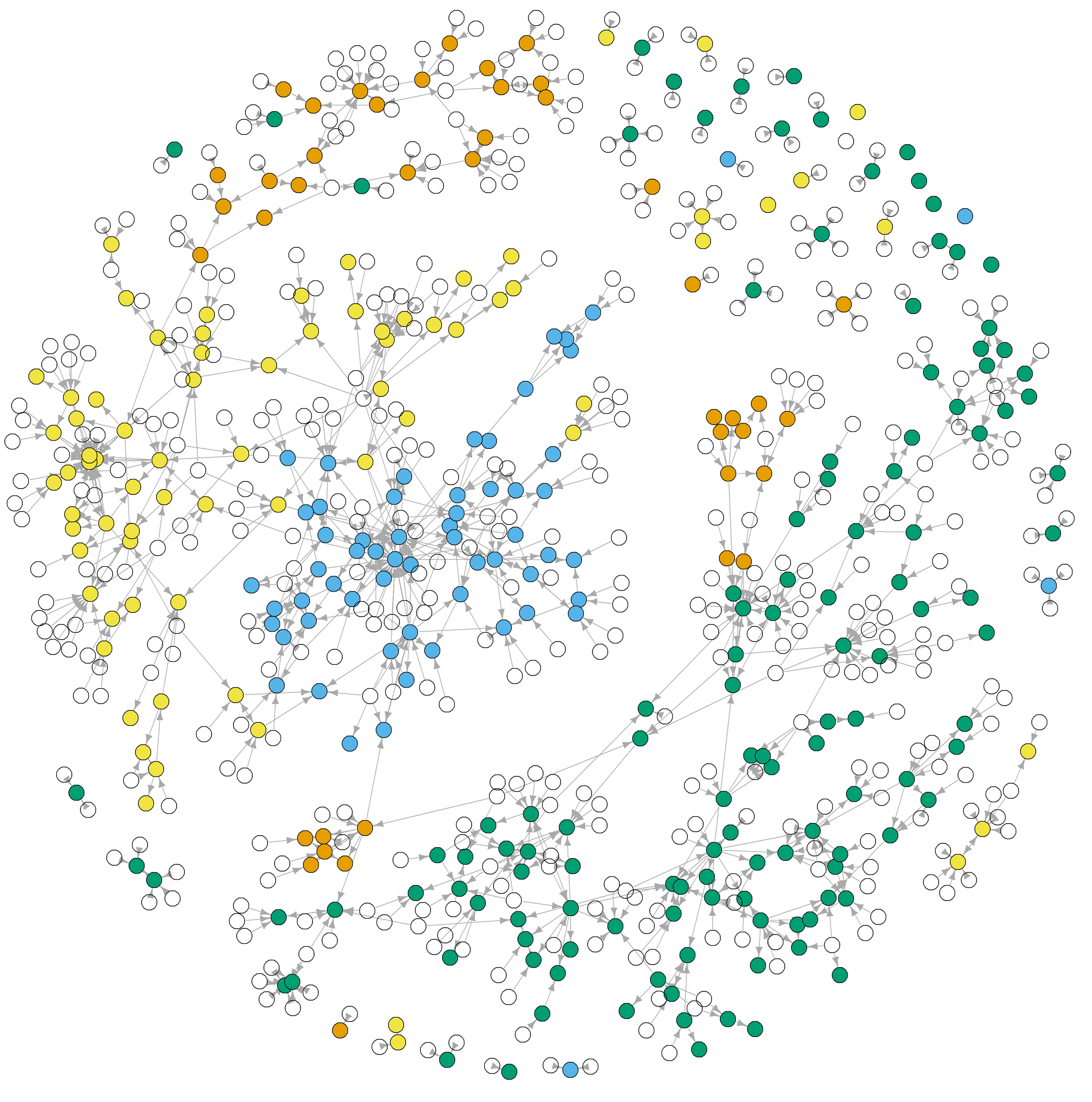

Supplement: S2 Fig — Dots representing participants are colored according to village (Binaoli = orange, Kolofi = green, Irobulu = blue, Lagoe = yellow). Dots representing people outside of the network (not interviewed because deceased or in other villages) are white. Arrows connecting dots represent flow of knowledge shared from one person to another. (TIF) [file pone.0239345.s002.tif]

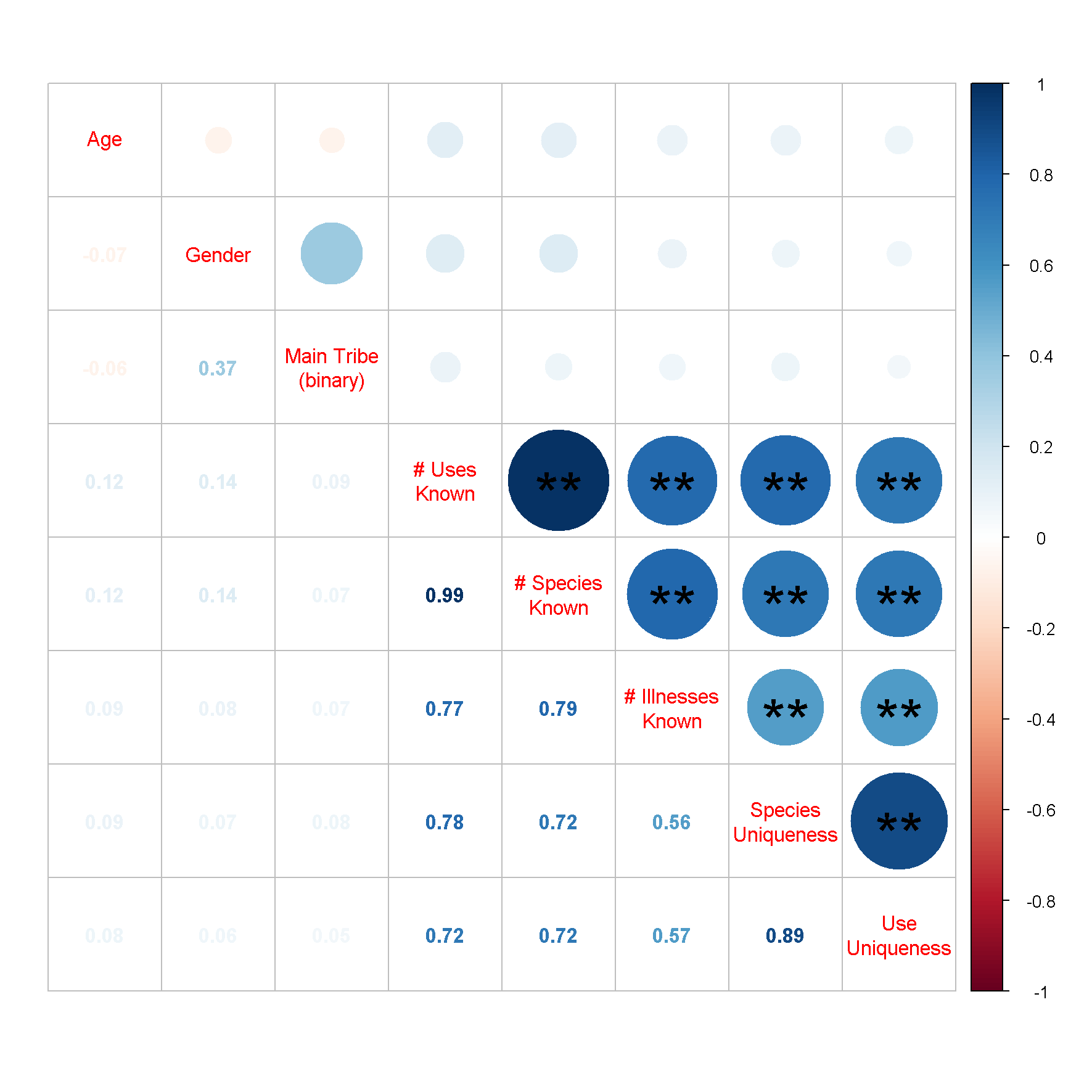

Supplement: S3 Fig — N = 303 people. Panels on the lower left display Pearson correlation coefficients. Panels on the upper right show circles whose size represents Pearson correlation coefficients. Weaker correlations are displayed in more transparent font and with smaller circles. Key: *p<0.1; **p<0.05; ***p<0.01. (TIF) [file pone.0239345.s003.tif]

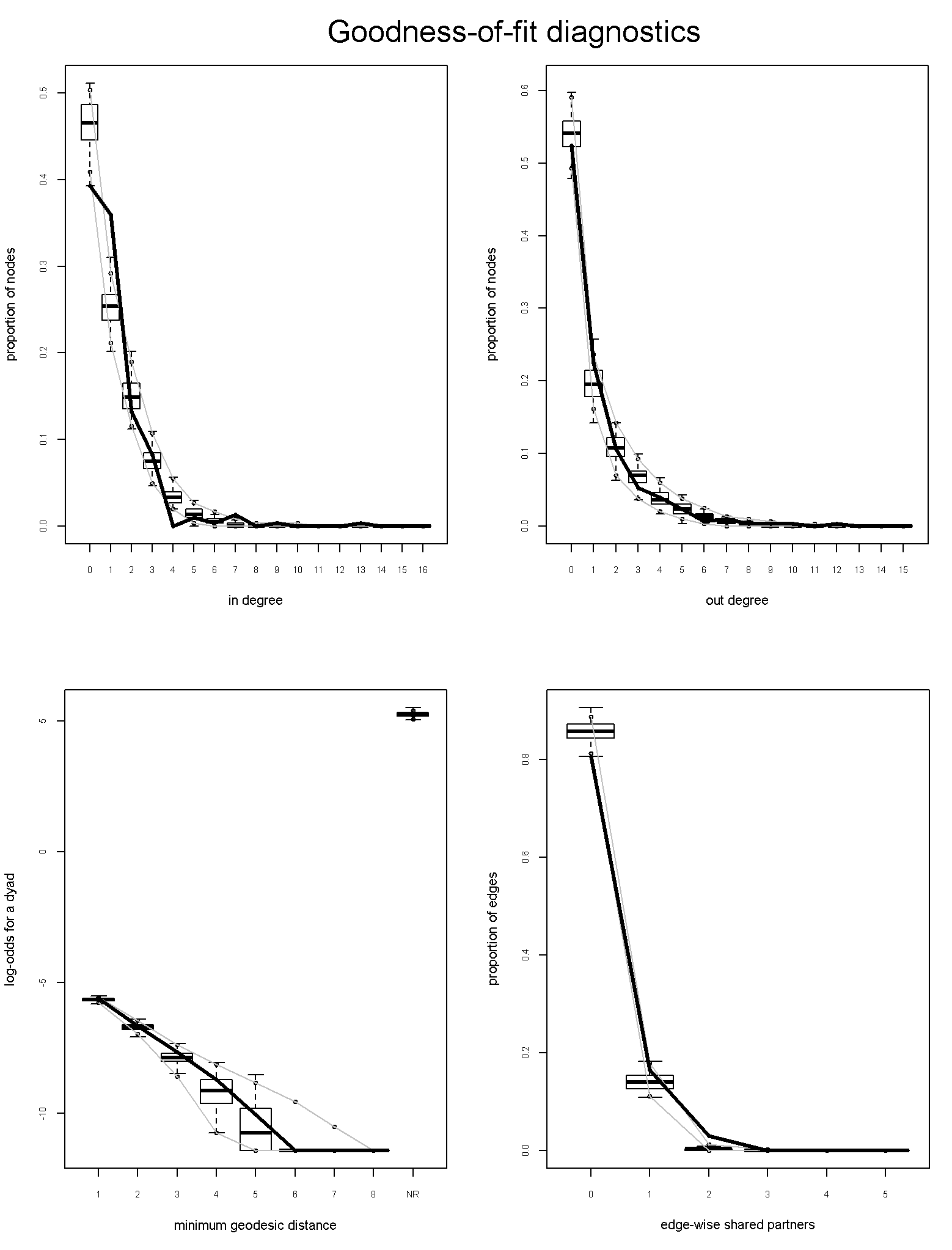

Supplement: S4 Fig — Solid lines represent the distributions of the observed statistics, and box plots summarize their distributions based on 100 simulated networks. (TIF) [file pone.0239345.s004.tif]

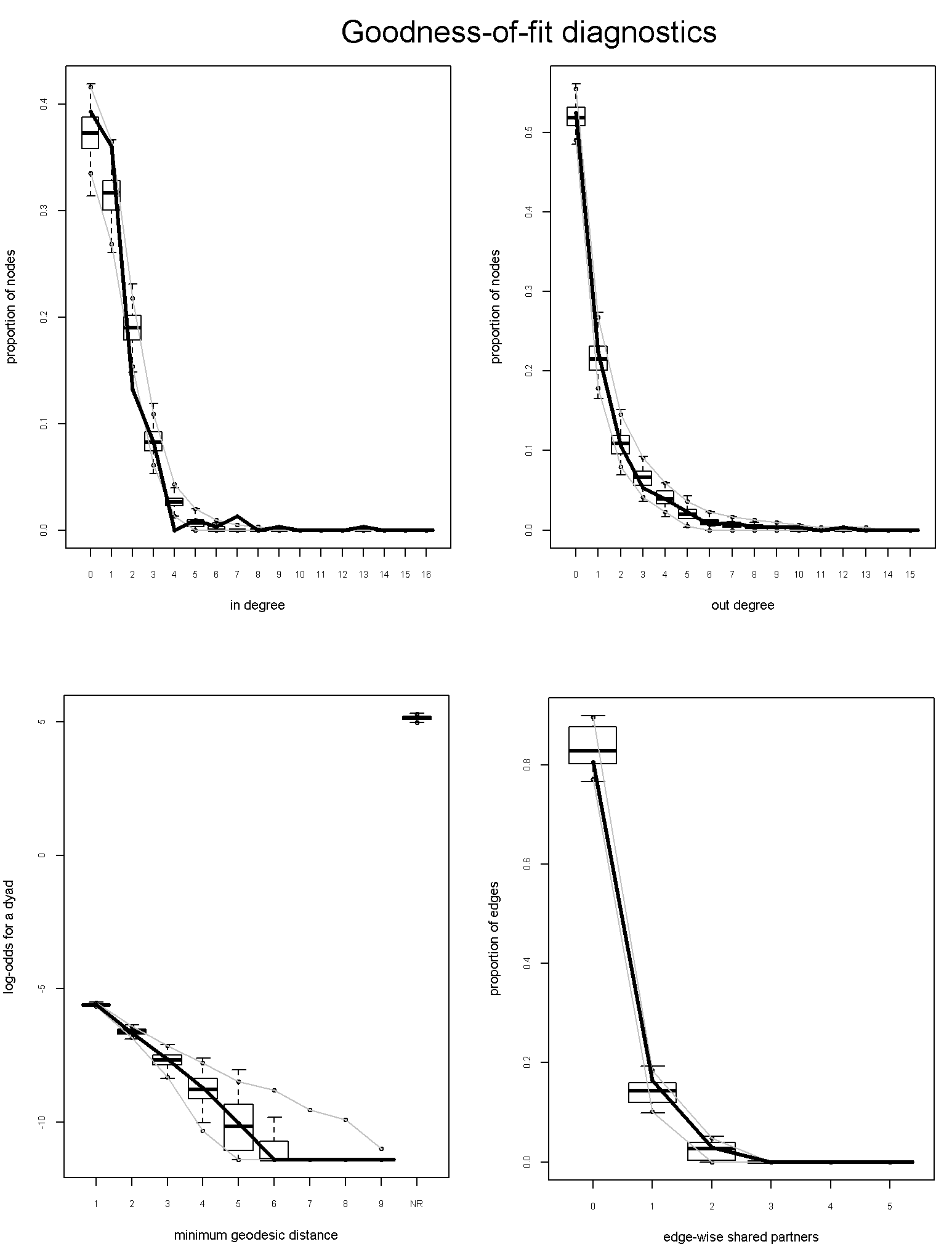

Supplement: S5 Fig — Solid lines represent the distributions of the observed statistics, and box plots summarize their distributions based on 100 simulated networks. (TIF) [file pone.0239345.s005.tif]

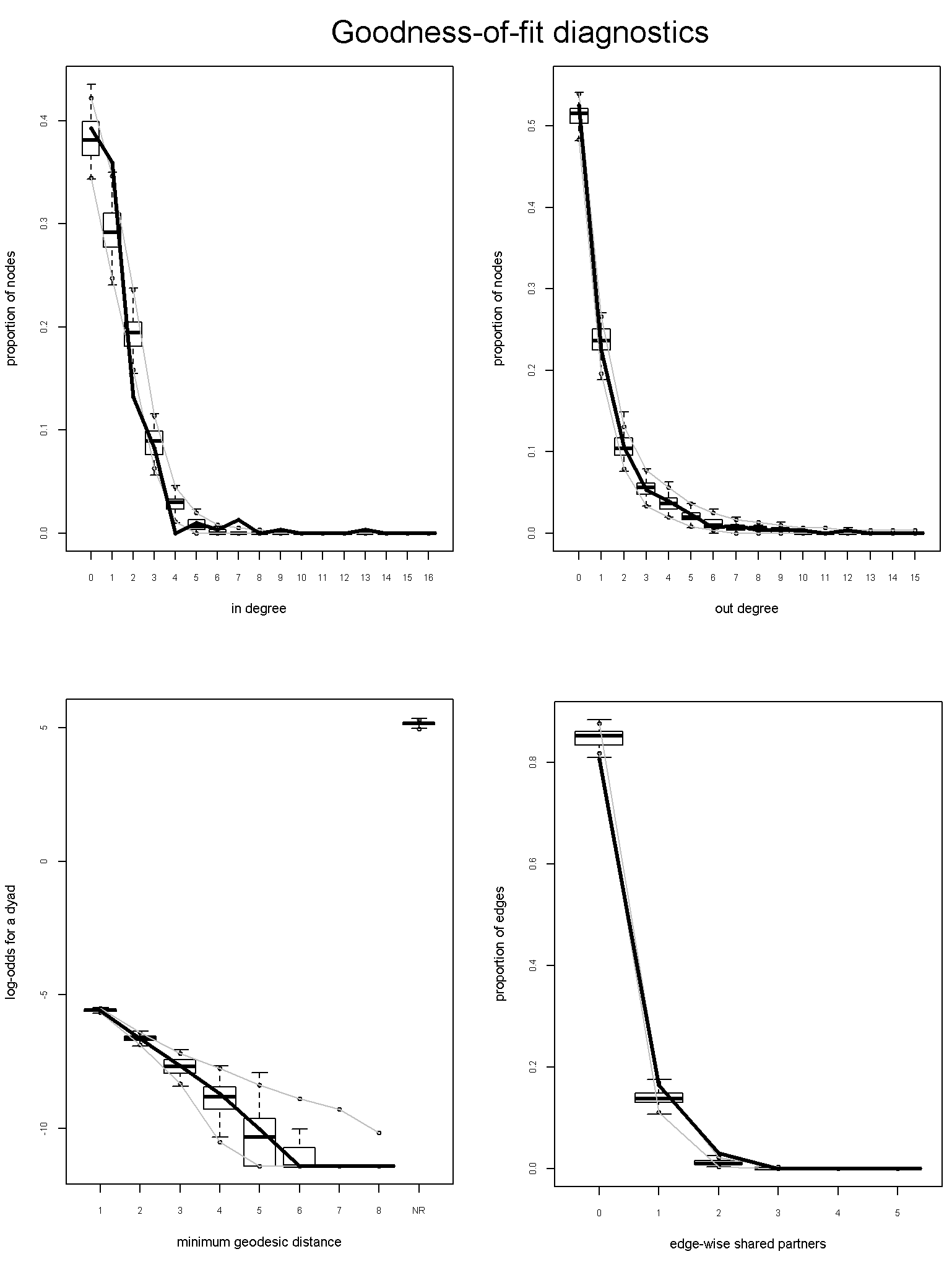

Supplement: S6 Fig — Solid lines represent the distributions of the observed statistics, and box plots summarize their distributions based on 100 simulated networks. (TIF) [file pone.0239345.s006.tif]

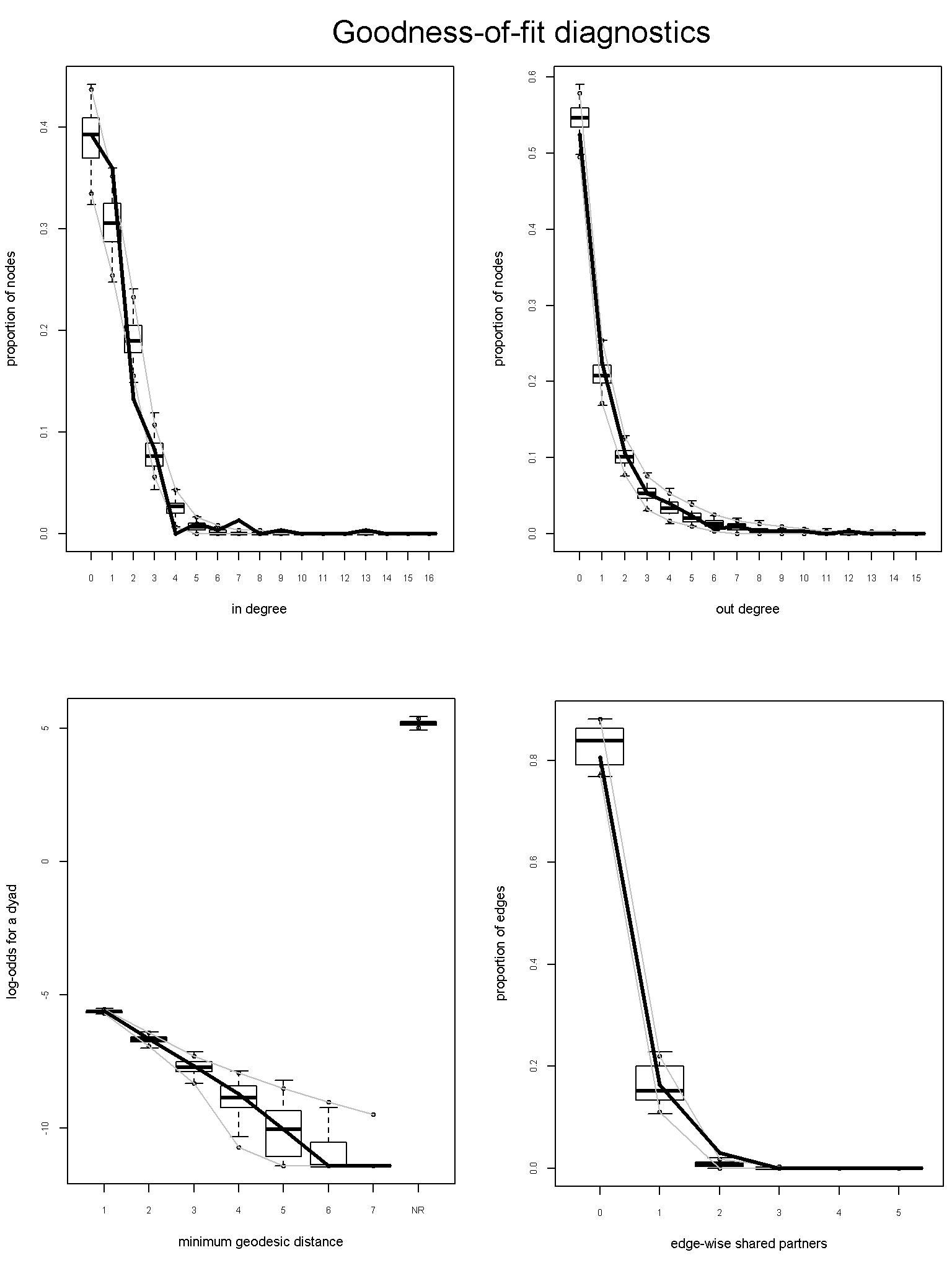

Supplement: S7 Fig — Solid lines represent the distributions of the observed statistics, and box plots summarize their distributions based on 100 simulated networks. (TIF) [file pone.0239345.s007.tif]

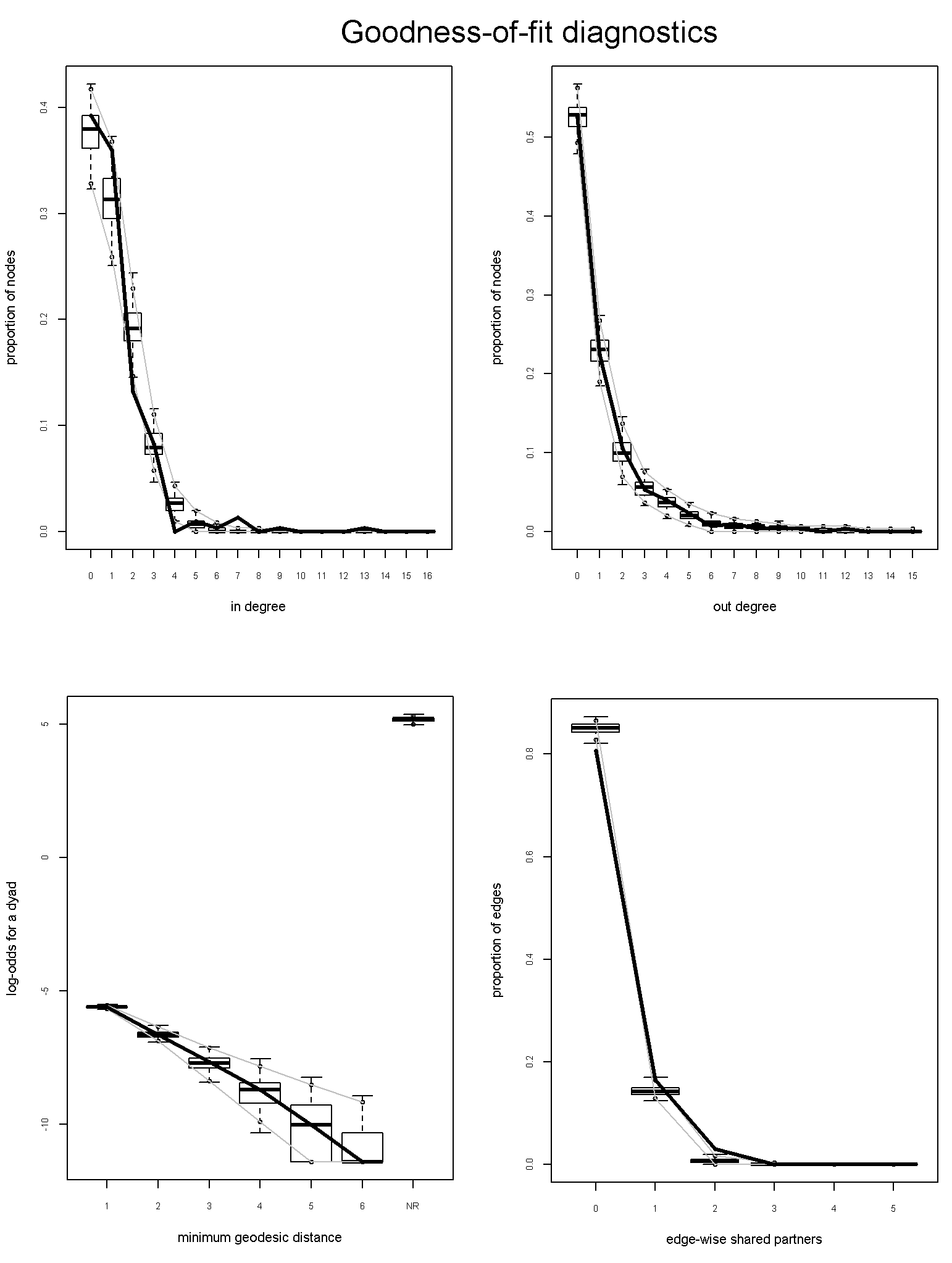

Supplement: S8 Fig — Solid lines represent the distributions of the observed statistics, and box plots summarize their distributions based on 100 simulated networks. (TIF) [file pone.0239345.s008.tif]

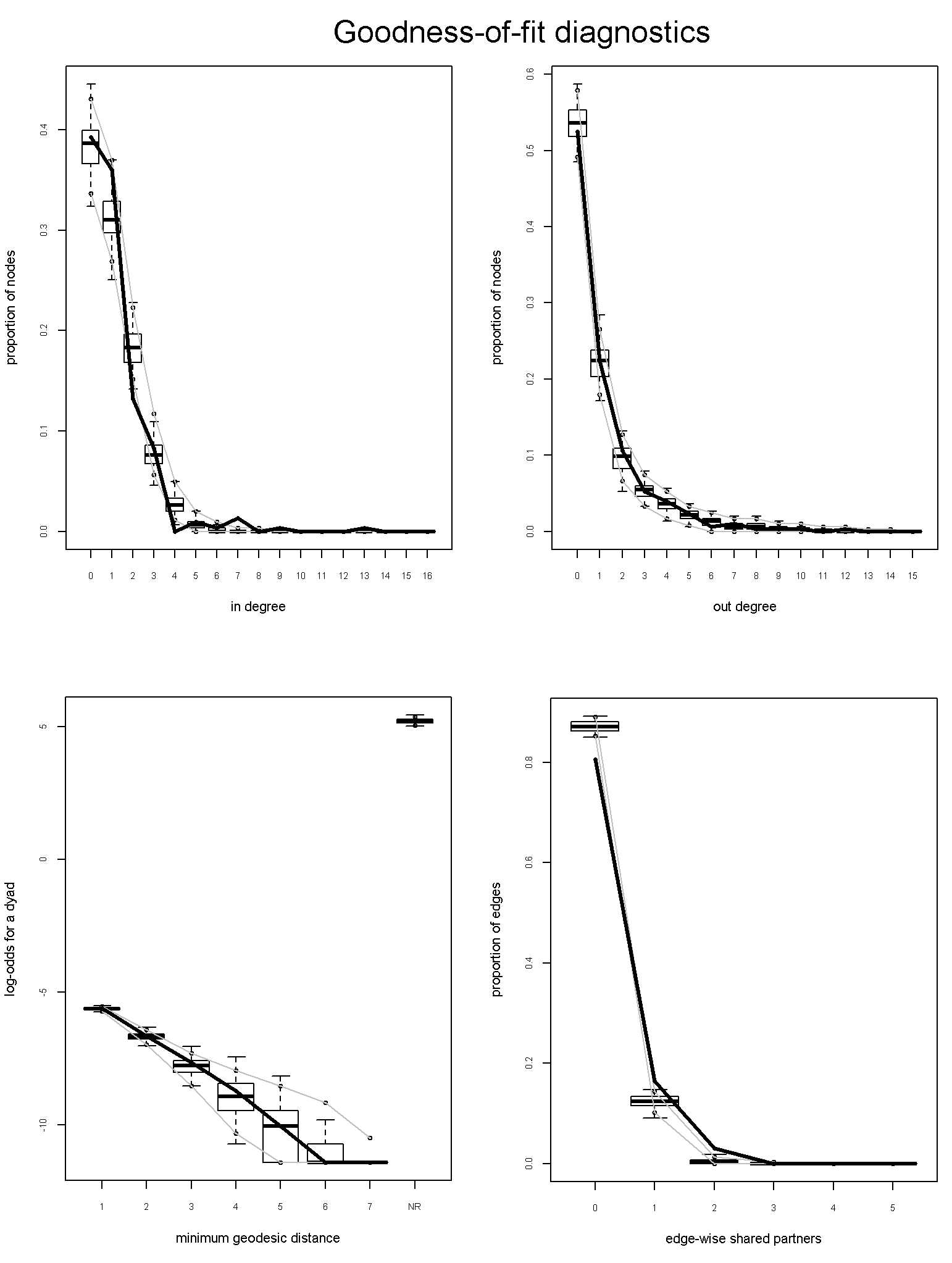

Supplement: S9 Fig — Solid lines represent the distributions of the observed statistics, and box plots summarize their distributions based on 100 simulated networks. (TIF) [file pone.0239345.s009.tif]

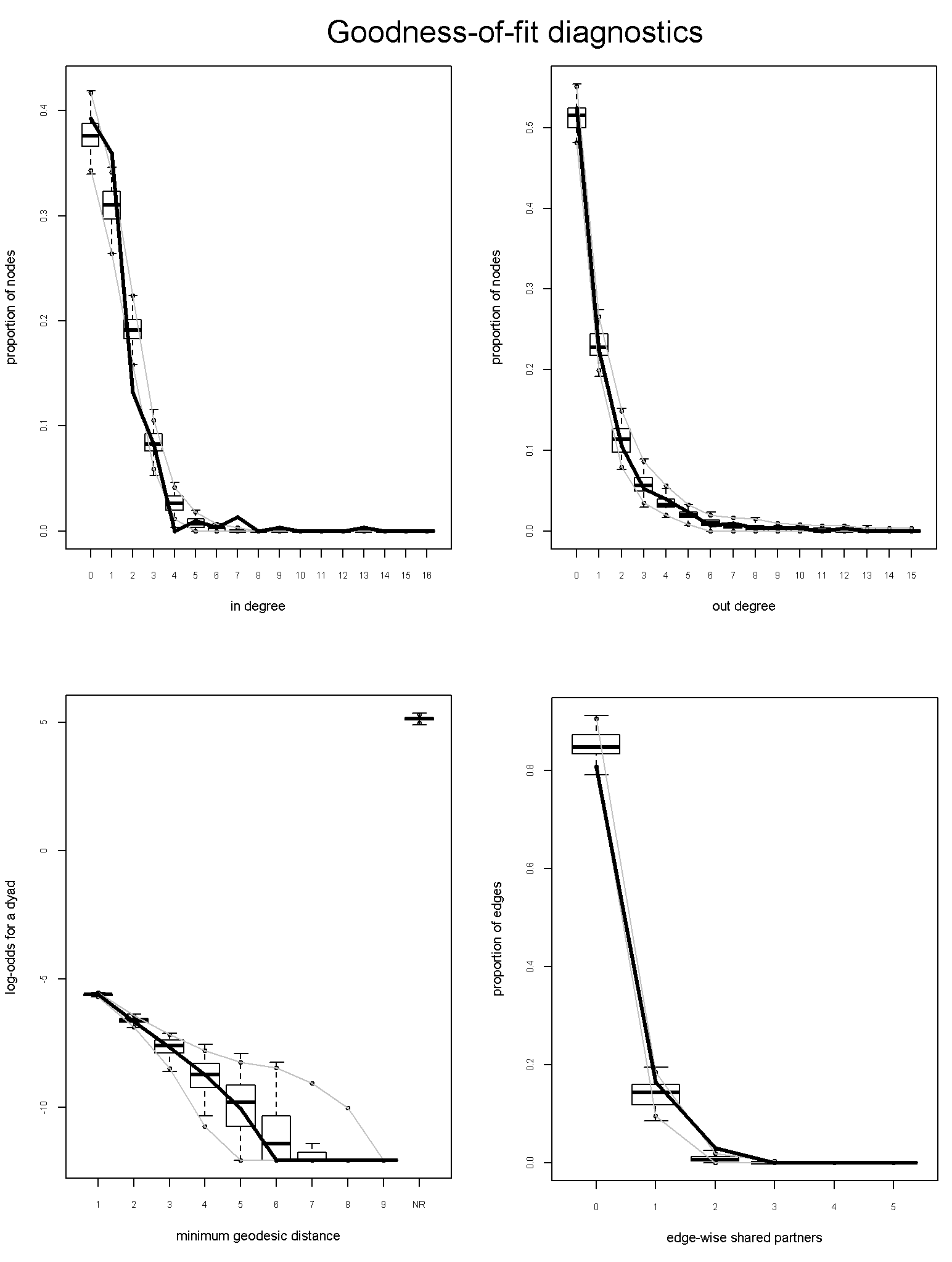

Supplement: S10 Fig — Solid lines represent the distributions of the observed statistics, and box plots summarize their distributions based on 100 simulated networks. (TIF) [file pone.0239345.s010.tif]

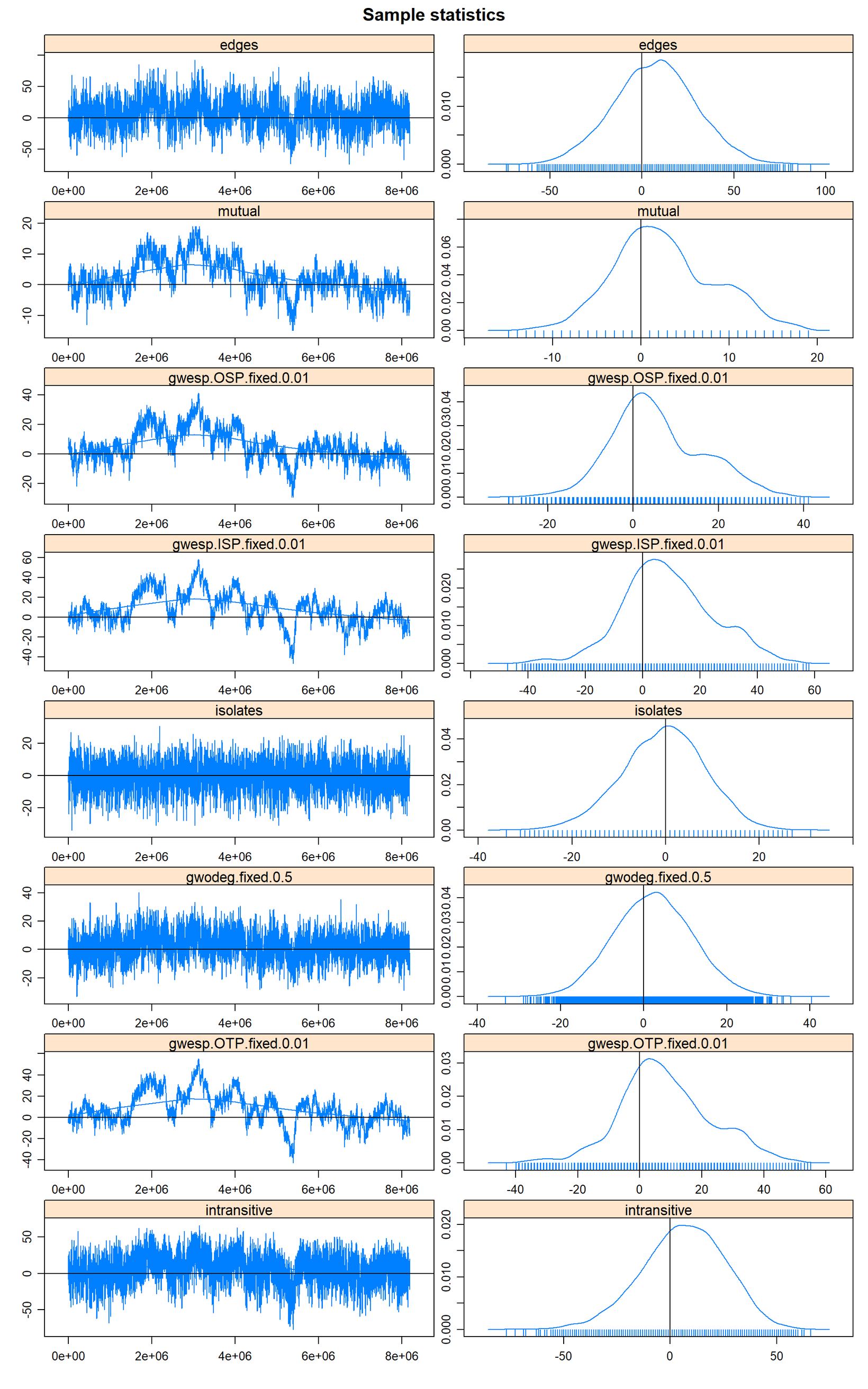

Supplement: S11 Fig — (TIF) [file pone.0239345.s011.tif]

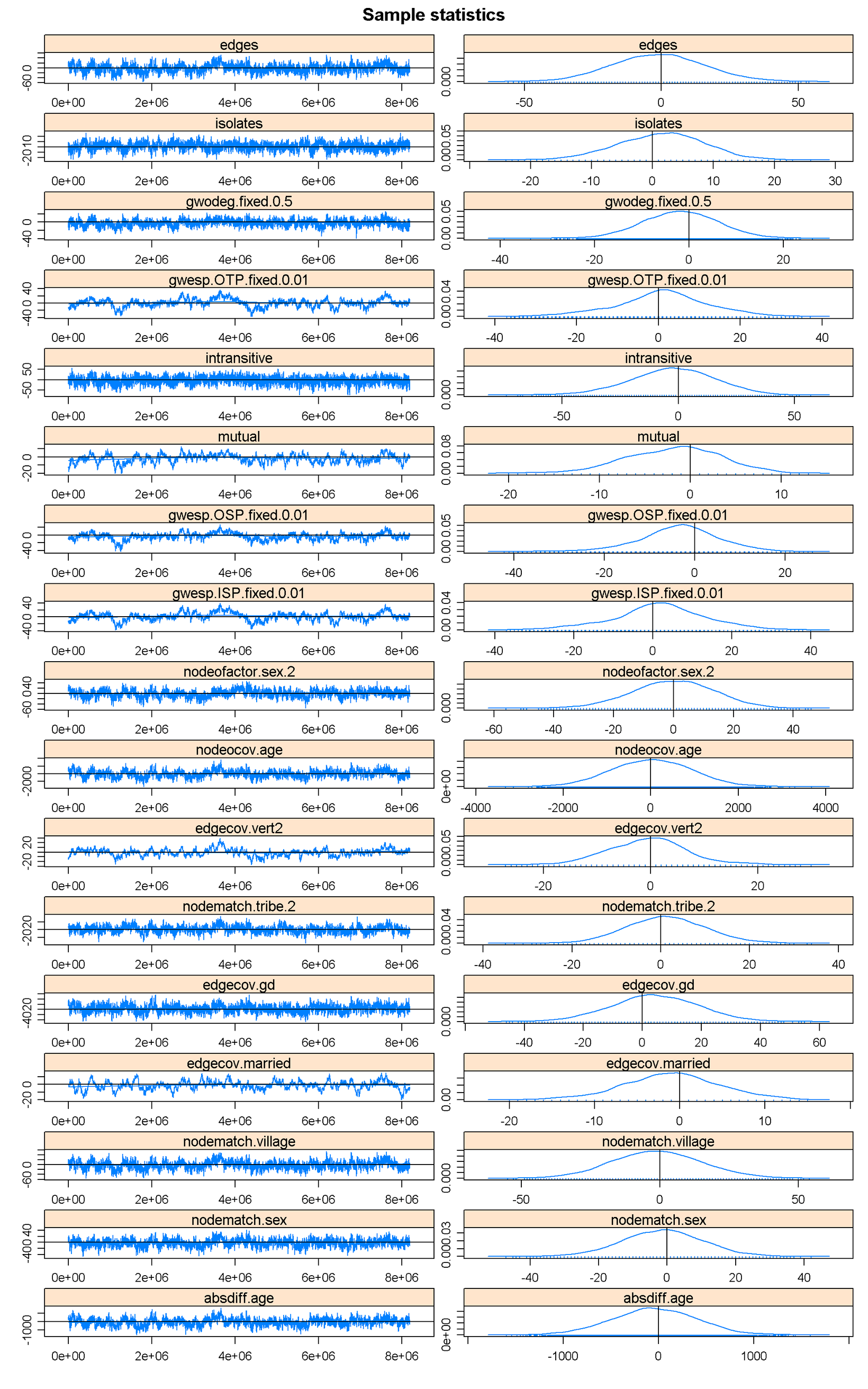

Supplement: S12 Fig — (TIF) [file pone.0239345.s012.tif]

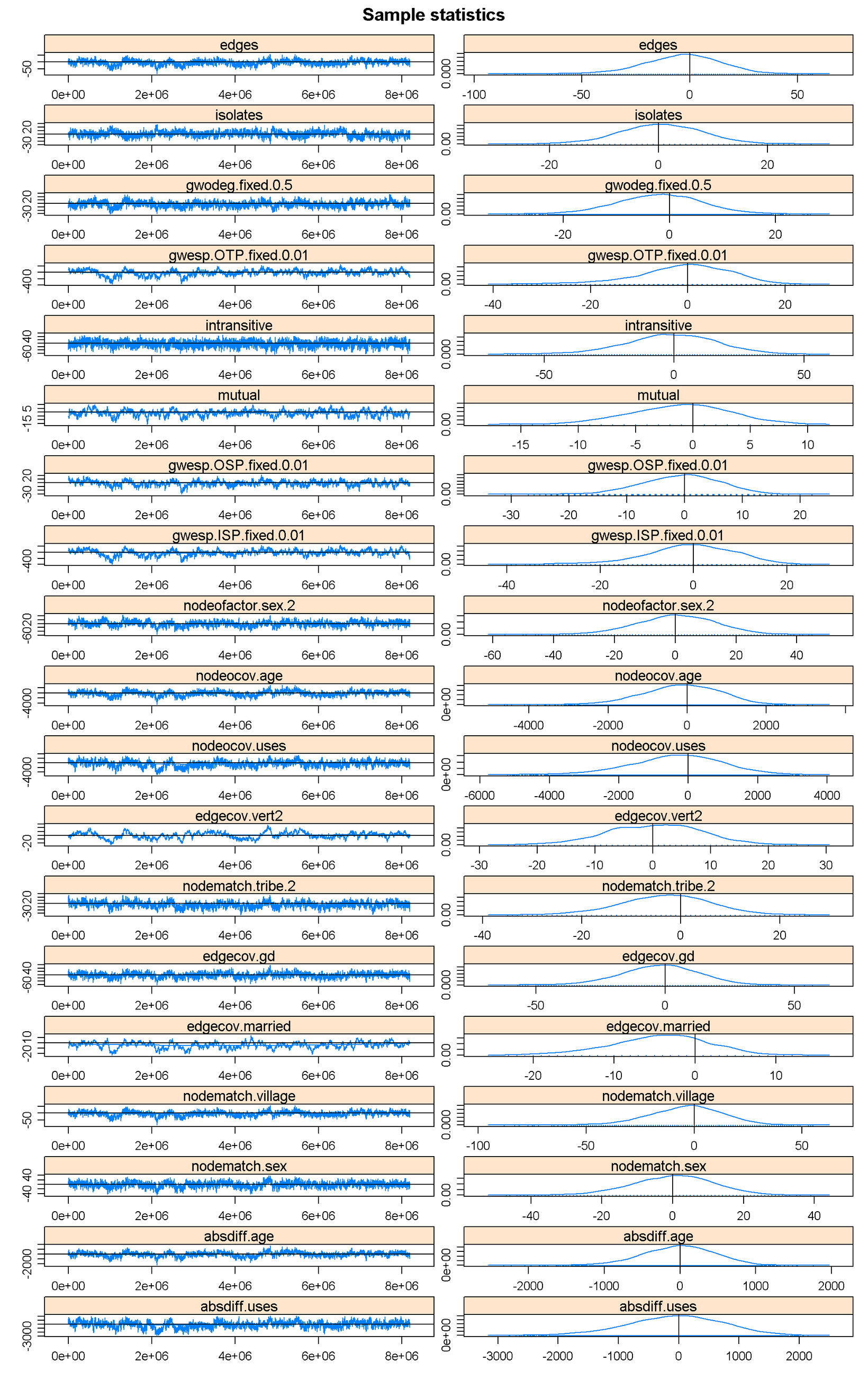

Supplement: S13 Fig — (TIF) [file pone.0239345.s013.tif]

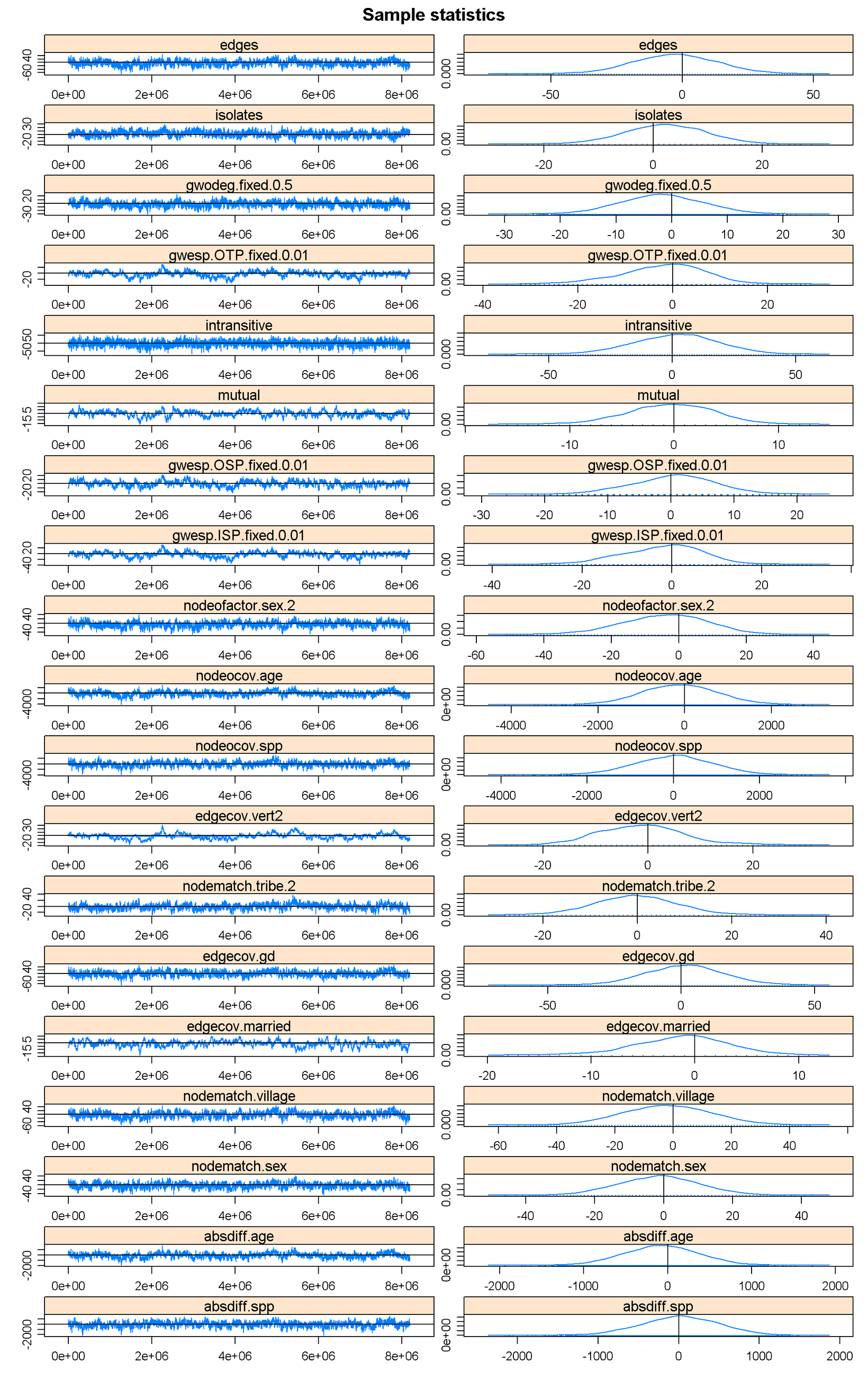

Supplement: S14 Fig — (TIF) [file pone.0239345.s014.tif]

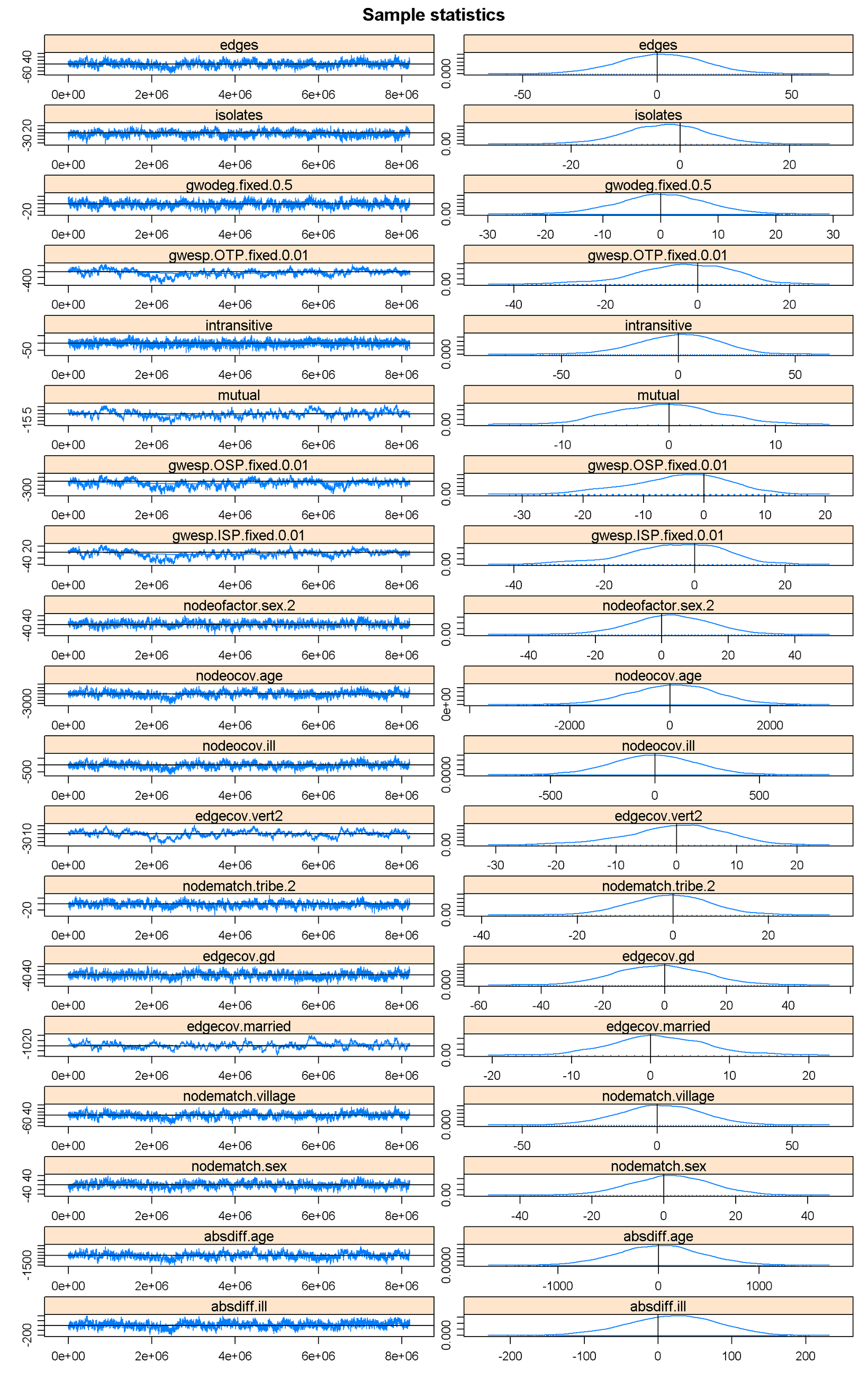

Supplement: S15 Fig — (TIF) [file pone.0239345.s015.tif]

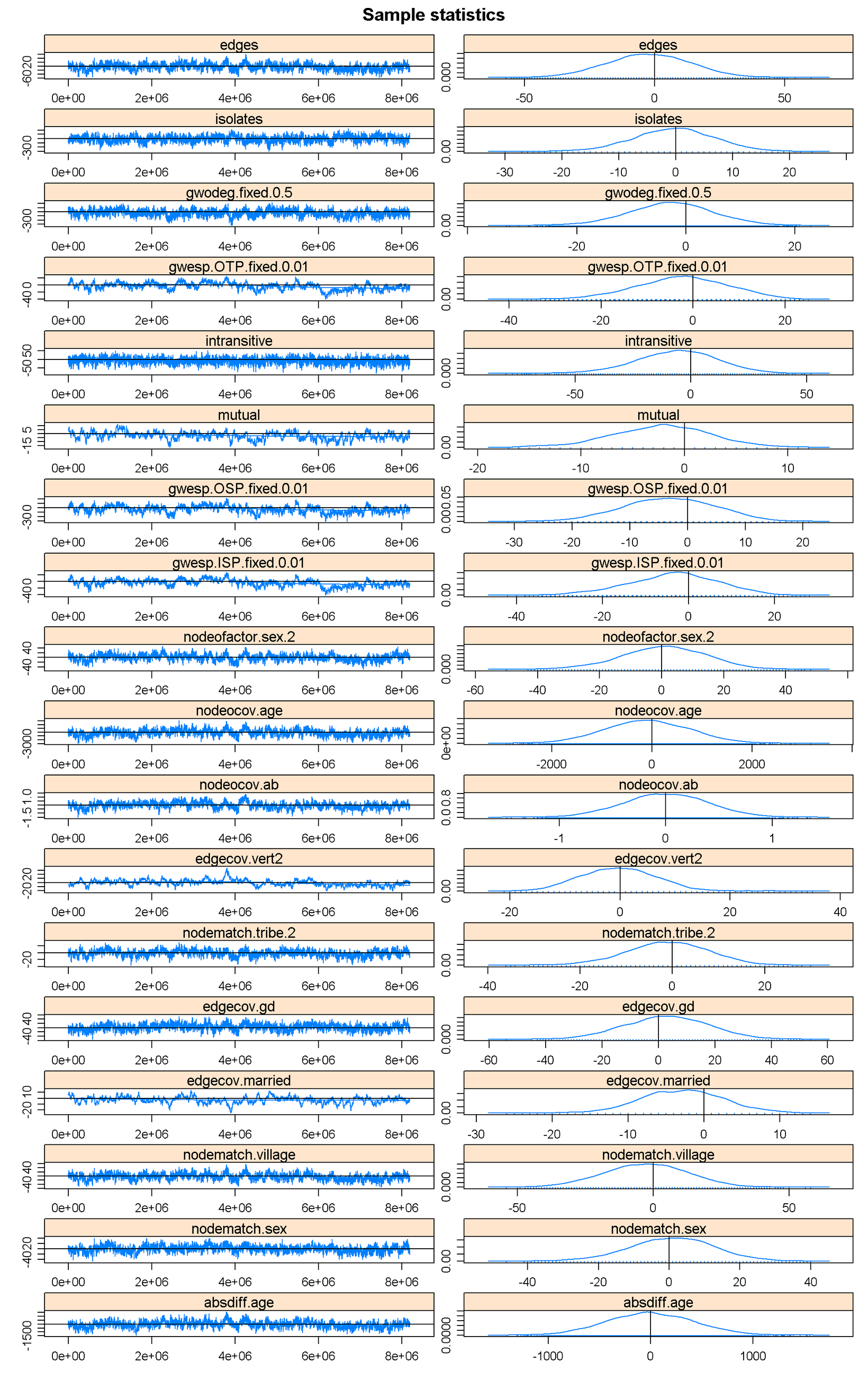

Supplement: S16 Fig — (TIF) [file pone.0239345.s016.tif]

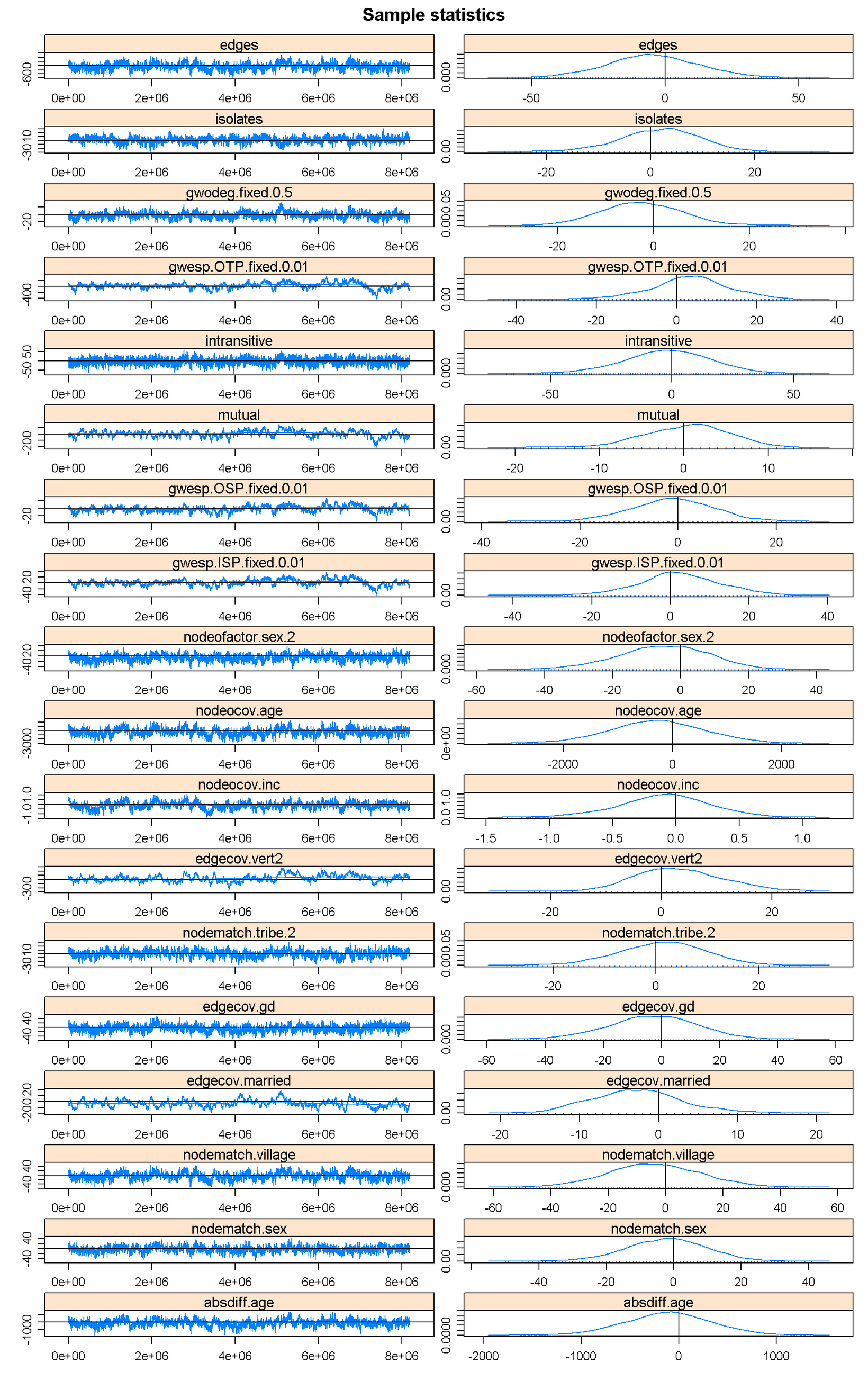

Supplement: S17 Fig — (TIF) [file pone.0239345.s017.tif]
