# Supplementary material for: Prestige and homophily predict network structure for social learning of medicinal plant knowledge
Source: PLoS One. 2020 Oct 8;15(10):e0239345. doi: 10.1371/journal.pone.0239345 (PMC7544085; doi:10.1371/journal.pone.0239345)
Supplement: S1 Table — (DOCX) [file pone.0239345.s018.docx]

**S1 Table. Summary information for closed network of research villages in North Malaita, Solomon Islands.**

|  | Binaoli | Kolofi | Irobulu | Lagoe |
| --- | --- | --- | --- | --- |
| # Participants/# eligible residents | 41/41 | 127/135 | 61/65 | 74/81 |
| Percent participation | 100% | 94% | 94% | 91% |
| # plant species in photograph checklist interview | 177 | 185 | 221 | 249 |
| Age (range, mean, standard deviation) | 18-73, 36.0, 14.4 | 18-74, 36.4, 14.6 | 18-76, 34.2, 14.2 | 18-75, 39.2, 16.0 |
| Gender (men) | 41% | 55% | 49% | 50% |
| Descendant of main kinship group (yes*) | 49% | 49% | 46% | 49% |
| Percent of participants who are spouses | 32% | 30% | 28% | 27% |
| # isolated nodes | 4 | 40 | 7 | 13 |
| Density (observed edges / possible edges) | 0.01042984 | 0.003877191 | 0.0124335 | 0.007323452 |
| Diameter (the largest number of connected edges) | 4 | 4 | 6 | 4 |
| Clustering (probability that nodes are part of triangles) | 0.1126005 | 0.09325246 | 0.08435852 | 0.1107784 |
| Centralization | 0.0521 | 0.0351 | 0.0639 | 0.0629 |
| In degree (range, mean, standard deviation) | 1-12, 2.0, 3.0 | 0-15, 2.4, 3.0 | 0-27, 3.6, 3.7 | 0-12, 3.3, 2.6 |
| Out degree (range, mean, standard deviation) | 0-7, 1.3, 1.8 | 0-12, 0.9, 1.7 | 0-10, 1.5, 2.0 | 0-9, 1.1, 1.7 |
| # uses cited per participant (range, mean, standard deviation) | 9-141, 53.2, 29.9 | 0-133, 36.0, 19.5 | 1-163, 63.0, 31.7 | 11-202, 57.0, 36.2 |
| # species cited per participant (range, mean, standard deviation) | 9-107, 44.2, 22.3 | 0-115, 31.2, 15.7 | 1-117, 52.0, 23.6 | 11-149, 47.0, 26.3 |
| # illness categories cited per participant (range, mean, standard deviation) | 5-17, 12.6, 3.3 | 0-16, 9.7, 2.9 | 1-17, 11.3, 3.1 | 5-18, 11.1, 2.8 |
| Uniqueness of uses known (range, mean, standard deviation) | 0.016-0.058, 0.024, 0.008 | 0.005-0.037, 0.008, 0.003 | 0.011-0.041, 0.016, 0.006 | 0.007-0.045, 0.014, 0.006 |
| Uniqueness of species known (range, mean, standard deviation) | 0.013-0.074, 0.024, 0.012 | 0.004-0.036, 0.008, 0.005 | 0.008-0.061, 0.016, 0.009 | 0.006-0.058, 0.014, 0.011 |

*indicates people who are descended from the single kinship group that is most common across all four villages. Remaining percent is composed of people descended from many different kinship groups
