# Supplementary material for: Prestige and homophily predict network structure for social learning of medicinal plant knowledge
Source: PLoS One. 2020 Oct 8;15(10):e0239345. doi: 10.1371/journal.pone.0239345 (PMC7544085; doi:10.1371/journal.pone.0239345)
Supplement: S1 File — (DOC) [file pone.0239345.s019.doc]

**S1 File. Interview Guides.**

**Interview Guide: Personal Interview 1:**

1. What is your name?
2. What plants you use medicinally?
3. For each plant that you use, what symptom(s) or disease(s) is it used for?
4. Where do you harvest these plants?
5. How old are you?
6. Do you ever go to health clinics or hospitals?
7. Where is the nearest health clinic or hospital?

**Interview Guide: Personal Interview 2:**

For each plant in photo list (say local name of plant while showing picture):

1. Do you use this plant as medicine? What symptom(s) or disease(s) is it used for?

After all photos have been shown:

1. How did you learn about medicinal plants?
2. Who taught you about medicinal plants (need their names)?
3. What is your relationship to each person who taught you, and where do they live?
