# Supplementary material for: Prestige and homophily predict network structure for social learning of medicinal plant knowledge
Source: PLoS One. 2020 Oct 8;15(10):e0239345. doi: 10.1371/journal.pone.0239345 (PMC7544085; doi:10.1371/journal.pone.0239345)
Supplement: S1 Appendix — (DOCX) [file pone.0239345.s020.docx]

**S1 Appendix. Medicinal Plant List – Alphabetical by Latin Name.**

| Family | Genus | Species |
| --- | --- | --- |
| Euphorbiaceae | *Acalypha* | *grandis* |
| Euphorbiaceae | *Acalypha* | *wilkesiana* |
| Orchidaceae | *Acriopsis* | *liliifolia* |
| Arecaceae | *Actinorhytis* | *calapparia* |
| Asteraceae | *Adenostemma* | *lavenia* |
| Fabaceae | *Adenanthera* | *pavonina* |
| Asteraceae | *Ageratum* | *conyzoides* |
| Meliaceae | *Aglaia* | *argentea* |
| Meliaceae | *Aglaia* | *brassii* |
| Polypodiaceae | *Aglaomorpha* | *drynarioides* |
| Orchidaceae | *Agrostophyllum* | *majus* |
| Araceae | *Alocasia* | *macrorrhizos* |
| Asphodelaceae | *Aloe* | *vera* |
| Zingiberaceae | *Alpinia* | *oceanica* |
| Zingiberaceae | *Alpinia* | *purpurata* |
| Apocynaceae | *Alstonia* | *scholaris* |
| Apocynaceae | *Alstonia* | *spectabilis* |
| Marattiaceae | *Angiopteris* | *microura* |
| Annonaceae | *Annona* | *muricata* |
| Moraceae | *Antiaris* | *toxicaria* |
| Fabaceae | *Archidendron* | *lucyi* |
| Fabaceae | *Archidendropsis* | *oblonga* |
| Arecaceae | *Areca* | *catachu* |
| Arecaceae | *Areca* | *macrocalyx* |
| Aristolochiaceae | *Aristolochia* | *acuminata* |
| Moraceae | *Artocarpus* | *altilis* |
| Asclepidaceae | *Asclepias* | *curassavica* |
| Rubiaceae | *Atractocarpus* | *macarthurii* |
| Chrysobalanaceae | *Atuna* | *racemosa* |
| Lecythidaceae | *Barringtonia* | *asiatica* |
| Begoniaceae | *Begonia* | *pinnatifida* |
| Begoniaceae | *Begonia* | *weigallii* |
| Phyllanthaceae | *Bischofia* | *javanica* |
| Asteraceae | *Blumea* | *riparia* |
| Asteraceae | *Blumea* | *sylvatica* |
| Urticaceae | *Boehmeria* | *celebica* |
| Brassicaceae | *Brassica* | *rapa* |
| Phyllanthaceae | *Breynia* | *cernua* |
| Rhizophoraceae | *Bruguiera* | *gymnorrhiza* |
| Rhizophoraceae | *Bruguiera* | *parviflora* |
| Crassulaceae | *Bryophyllum* | *pinnatum* |
| Lamiaceae | *Callicarpa* | *pentandra* |
| Anacardiaceae | *Campnosperma* | *brevipetiolatum* |
| Burseraceae | *Canarium* | *indicum* |
| Burseraceae | *Canarium* | *lamii* |
| Annonaceae | *Cananga* | *odorata* |
| Fabaceae | *Canavalia* | *rosea* |
| Solanaceae | *Capsicum* | *annuum* |
| Caricaceae | *Carica* | *papaya* |
| Apocynaceae | *Carruthersia* | *pilosa* |
| Arecaceae | *Caryota* | *rumphiana* |
| Salicaceae | *Casearia* | *clutiifolia* |
| Salicaceae | *Casearia* | *grewiifolia* |
| Fabaceae | *Cassia* | *alata* |
| Cannabaceae | *Celtis* | *latifolia* |
| Poaceae | *Centotheca* | *lappacea* |
| Apocynaceae | *Cerbera* | *manghas* |
| Costaceae | *Cheilocostus* | *specosius* |
| Thelypteridaceae | *Christella* | *dentata* |
| Lauraceae | *Cinnamomum* | *solomonense* |
| Cucurbitaceae | *Citrullus* | *lanatus* |
| Rutaceae | *Citrus* | *maxima* |
| Rutaceae | *Citrus* | *xlimon* |
| Rutaceae | *Citrus* | *xlimon* |
| Euphorbiaceae | *Claoxylon* | *tumidum* |
| Ranunculaceae | *Clematis* | *pickeringii* |
| Lamiaceae | *Clerodendrum* | *buchananii* |
| Lamiaceae | *Clerodendrum* | *buchananii* |
| Lamiaceae | *Clerodendrum* | *quadriloculare* |
| Melastomataceae | *Clidemia* | *hirta* |
| Arecaceae | *Cocos* | *nucifera* |
| Euphorbiaceae | *Codiaeum* | *variegatum* |
| Euphorbiaceae | *Codiaeum* | *variegatum* |
| Araceae | *Colocasia* | *esculenta* |
| Malvaceae | *Commersonia* | *bartramia* |
| Connaraceae | *Connarus* | *salomoniensis* |
| Asparagaceae | *Cordyline* | *fruticosa* |
| Asparagaceae | *Cordyline* | *fruticosa* |
| Asteraceae | *Crassocephalum* | *crepidioides* |
| Capparaceae | *Crateva* | *religiosa* |
| Amaryllidaceae | *Crinum* | *asiaticum* |
| Cucurbitaceae | *Cucumis* | *sativus* |
| Zingiberaceae | *Curcuma* | *longa* |
| Cycadaceae | *Cycas* | *rumphii* |
| Poaceae | *Cymobopogon* | *citratus* |
| Gesneriaceae | *Cyrtandra* | *filibracteata* |
| Loranthaceae | *Dactyliophora* | *salomonia* |
| Davalliaceae | *Davallia* | *solida* |
| Fabaceae | *Delonix* | *regia* |
| Urticaceae | *Dendrocnide* | *kajewskii* |
| Fabaceae | *Derris* | *elliptica* |
| Fabaceae | *Derris* | *scandens* |
| Fabaceae | *Desmodium* | *incanum* |
| Fabaceae | *Desmodium* | *triflorum* |
| Fabaceae | *Desmodium* | *umbellatum* |
| Dichapetalaceae | *Dichapetalum* | *papuanum* |
| Araceae | *Dieffenbachia* | *amoena* |
| Dioscoreaceae | *Dioscorea* | *bulbifera* |
| Athyriaceae | *Diplazium* | *esculentum* |
| Rubiaceae | *Dolicholobium* | *acuminatum* |
| Rubiaceae | *Dolicholobium* | *glabrum* |
| Rubiaceae | *Dolicholobium* | *solomonense* |
| Marantaceae | *Donax* | *canniformis* |
| Asparagaceae | *Dracaena* | *angustifolia* |
| Meliaceae | *Dysoxylum* | *dolichobotrys* |
| Meliaceae | *Dysoxylum* | *kaniense* |
| Meliaceae | *Dysoxylum* | *parasiticum* |
| Urticaceae | *Elatostema* | *sesquifolium* |
| Poaceae | *Eleusine* | *indica* |
| Fabaceae | *Entada* | *phaceoloides* |
| Fabaceae | *Erythrina* | *variegata* |
| Myrtaceae | *Eucalyptus* | *deglupta* |
| Rutaceae | *Euodia* | *hortensis* |
| Rutaceae | *Euodia* | *whitmorei* |
| Euphorbiaceae | *Euphorbia* | *heterophylla* |
| Euphorbiaceae | *Euphorbia* | *hirta* |
| Euphorbiaceae | *Euphorbia* | *tithymaloides* |
| Loganiaceae | *Fagraea* | *racemosa* |
| Fabaceae | *Falcataria* | *moluccana* |
| Moraceae | *Ficus* | *adenosperma* |
| Moraceae | *Ficus* | *austrina* |
| Moraceae | *Ficus* | *benjamina* |
| Moraceae | *Ficus* | *copiosa* |
| Moraceae | *Ficus* | *cristobalensis* |
| Moraceae | *Ficus* | *endochaete* |
| Moraceae | *Ficus* | *longibracteata* |
| Moraceae | *Ficus* | *nodosa* |
| Moraceae | *Ficus* | *oleracea* |
| Moraceae | *Ficus* | *pseudowassa* |
| Moraceae | *Ficus* | *septica* |
| Moraceae | *Ficus* | *smithii* |
| Moraceae | *Ficus* | *storckii* |
| Moraceae | *Ficus* | *subulata* |
| Moraceae | *Ficus* | *tinctoria* |
| Moraceae | *Ficus* | *trachypison* |
| Moraceae | *Ficus* | *wassa* |
| Flagellariaceae | *Flagellaria* | *gigantea* |
| Flagellariaceae | *Flagellaria* | *indica* |
| Pandanaceae | *Freycinetia* | *divericata* |
| Clusiaceae | *Garcinia* | *platyphylla* |
| Clusiaceae | *Garcinia* | *sessilis* |
| Rubiaceae | *Geophila* | *repens* |
| Phyllanthaceae | *Glochidion* | *angulatum* |
| Phyllanthaceae | *Glochidion* | *ferdinandi* |
| Orchidaceae | *Grammatophyllum* | *scriptum* |
| Halimedaceae | *Halimeda* | *macroloba* |
| Pteridaceae | *Haplopteris* | *ensiformis* |
| Acanthaceae | *Hemigraphis* | *alternata* |
| Acanthaceae | *Hemigraphis* | *reptans* |
| Malvaceae | *Heritiera* | *solomonensis* |
| Hernandiaceae | *Hernandia* | *guianensis* |
| Malvaceae | *Hibiscus* | *rosa-sinensis* |
| Malvaceae | *Hibiscus* | *tilliaceus* |
| Araceae | *Homalomena* | *melanesica* |
| Euphorbiaceae | *Homalanthus* | *nutans* |
| Zingiberaceae | *Hornstedtia* | *scottiana* |
| Myristicaceae | *Horsfieldia* | *solomonensis* |
| Myristicaceae | *Horsfieldia* | *whitmorei* |
| Asclepidaceae | *Hoya* | *cominsii* |
| Arecaceae | *Hydriastele* | *macrospadix* |
| Balsaminaceae | *Impatiens* | *balsamina* |
| Fabaceae | *Inocarpus* | *fagifer* |
| Fabaceae | *Intsia* | *bijuga* |
| Convolvulaceae | *Ipomoea* | *batatas* |
| Convolvulaceae | *Ipomoea* | *indica* |
| Convolvulaceae | *Ipomoea* | *pescaprae* |
| Convolvulaceae | *Ipomoea* | *quamoclit* |
| Crassulaceae | *Kalanchoe* | *blossfeldiana* |
| Monimiaceae | *Kibara* | *coriacea* |
| Malvaceae | *Kleinhovia* | *hospita* |
| Verbenaceae | *Lantana* | *camara* |
| Vitaceae | *Leea* | *tetramera* |
| Arecaceae | *Licuala* | *lauterbachii* |
| Lauraceae | *Litsea* | *domarensis* |
| Lauraceae | *Litsea* | *perglabra* |
| Lophopyxidaceae | *Lophopyxis* | *maingayi* |
| Onagraceae | *Ludwigia* | *octovalvis* |
| Euphorbiaceae | *Macaranga* | *aleuritoides* |
| Euphorbiaceae | *Macaranga* | *doica* |
| Euphorbiaceae | *Macaranga* | *fimbriata* |
| Euphorbiaceae | *Macaranga* | *magnifolia* |
| Euphorbiaceae | *Macaranga* | *tanarius* |
| Euphorbiaceae | *Macaranga* | *whitmorei* |
| Primulaceae | *Maesa* | *haplobotrys* |
| Euphorbiaceae | *Mallotus* | *mollissimus* |
| Anacardiaceae | *Mangifera* | *indica* |
| Anacardiaceae | *Mangifera* | *minor* |
| Euphorbiaceae | *Manihot* | *esculenta* |
| Chrysobalanaceae | *Maranthes* | *corymbosa* |
| Melastomataceae | *Melastoma* | *malabathricum* |
| Rutaceae | *Melicope* | *burttiana* |
| Rutaceae | *Melicope* | *burttiana* |
| Rutaceae | *Melicope* | *elleryana* |
| Rutaceae | *Melicope* | *latifolia* |
| Malvaceae | *Melochia* | *umbellata* |
| Convolvulaceae | *Merremia* | *peltata* |
| Rutaceae | *Micromelum* | *minutum* |
| Asteraceae | *Mikania* | *cordata* |
| Rubiaceae | *Morinda* | *citrifolia* |
| Fabaceae | *Mucuna* | *gigantea* |
| Musaceae | *Musa* | *maclayi* |
| Musaceae | *Musa* | *xparadisiaca* |
| Rubiaceae | *Mussaenda* | *cylindrocarpa* |
| Rubiaceae | *Mussaenda* | *kajewskii* |
| Myristicaceae | *Myristica* | *fatua* |
| Poaceae | *Nastus* | *obtusus* |
| Solanaceae | *Nicotiana* | *tabacum* |
| Urticaceae | *Nothocnide* | *repanda* |
| Nymphalidaceae | *Nymphaea* | *pubescens* |
| Arecaceae | *Nypa* | *fruticans* |
| Lamiaceae | *Ocimum* | *gratissimum* |
| Lamiaceae | *Ocimum* | *tenuiflorum* |
| Acanthaceae | *Odontonema* | *tubaeforme* |
| Ophioglossaceae | *Ophioderma* | *pendulum* |
| Araliaceae | *Osmoxylon* | *puniceopolleniferum* |
| Araliaceae | *Osmoxylon* | *tetrandrum* |
| Oxalidaceae | *Oxalis* | *exilis* |
| Pandanaceae | *Pandanus* | *compressus* |
| Pandanaceae | *Pandanus* | *dubius* |
| Achariaceae | *Pangium* | *edule* |
| Apocynaceae | *Parabaena* | *tuberculata* |
| Moraceae | *Paratocarpus* | *venenosa* |
| Poaceae | *Paspalum* | *conjugatum* |
| Passifloraceae | *Passiflora* | *foetida* |
| Poaceae | *Pennisetum* | *macrostachyum* |
| Phyllanthaceae | *Phyllanthus* | *amarus* |
| Phyllanthaceae | *Phyllanthus* | *ciccoides* |
| Solanaceae | *Physalis* | *angulata* |
| Urticaceae | *Pilea* | *microphylla* |
| Euphorbiaceae | *Pimelodendron* | *amboinicum* |
| Piperaceae | *Piper* | *betle* |
| Piperaceae | *Piper* | *bosnicanum* |
| Piperaceae | *Piper* | *macropiper* |
| Piperaceae | *Piper* | *sclerophloeum* |
| Piperaceae | *Piper* | *wichmanii* |
| Urticaceae | *Pipturus* | *argenteus* |
| Sapotaceae | *Planchonella* | *chartacea* |
| Sapotaceae | *Planchonella* | *myrsinodendron* |
| Lamiaceae | *Plectranthus* | *scutellarioides* |
| Dryopteridaceae | *Pleocnemia* | *irregularis* |
| Podocarpaceae | *Podocarpus* | *salomoniensis* |
| Araliaceae | *Polyscias* | *macgillivrayi* |
| Polygalaceae | *Polygala* | *paniculata* |
| Sapindaceae | *Pometia* | *pinnata* |
| Fabaceae | *Pongamia* | *pinnata* |
| Lamiaceae | *Premna* | *corymbosa* |
| Urticaceae | *Procris* | *frutescens* |
| Acanthaceae | *Pseuderanthemum* | *bibracteatum* |
| Acanthaceae | *Pseuderanthemum* | *whartonianum* |
| Myrtaceae | *Psidium* | *guajava* |
| Rubiaceae | *Psydrax* | *cymigera* |
| Fabaceae | *Pterocarpus* | *indicus* |
| Arecaceae | *Ptychosperma* | *salomonense* |
| Fabaceae | *Pueraria* | *montana* |
| Fabaceae | *Pueraria* | *phaseoloides* |
| Polypodiaceae | *Pyrrosia* | *lanceolata* |
| Anacardiaceae | *Rhus* | *taitensis* |
| Violaceae | *Rinorea* | *horneri* |
| Rosaceae | *Rubus* | *dendrocharis* |
| Poaceae | *Saccharum* | *officinarum* |
| Acanthaceae | *Sanchezia* | *parvibracteata* |
| Asparagaceae | *Sansevieria* | *trifasciata* |
| Actinidiaceae | *Saurauia* | *conferta* |
| Goodeniaceae | *Scaevola* | *taccada* |
| Araliaceae | *Schefflera* | *bougainvilleana* |
| Araliaceae | *Schefflera* | *stahliana* |
| Araceae | *Schismatoglottis* | *calyptrata* |
| Schizeaceae | *Schizea* | *dichotoma* |
| Poaceae | *Schizostachyum* | *tessellatum* |
| Fabaceae | *Schleinitzia* | *novo-guineensis* |
| Araceae | *Scindapsus* | *altissimus* |
| Phyllanthaceae | *Securinega* | *flexuosa* |
| Selaginellaceae | *Selaginella* | *rechingeri* |
| Anacardiaceae | *Semecarpus* | *brachystachys* |
| Fabaceae | *Serianthes* | *hooglandii* |
| Malvaceae | *Sida* | *rhombifolia* |
| Smilacaceae | *Smilax* | *extensa* |
| Fabaceae | *Sophora* | *tomentosa* |
| Orchidaceae | *Spathoglottis* | *plicata* |
| Cyatheaceae | *Sphaeropteris* | *brackenridgei* |
| Anacardiaceae | *Spondias* | *dulcis* |
| Verbenaceae | *Stachytarpheta* | *cayennensis* |
| Menispermaceae | *Stephania* | *japonica* |
| Malvaceae | *Sterculia* | *parkinsonii* |
| Malvaceae | *Sterculia* | *shillinglawii* |
| Moraceae | *Streblus* | *glaber* |
| Asteraceae | *Synedrella* | *nodiflora* |
| Myrtaceae | *Syzygium* | *aqueum* |
| Myrtaceae | *Syzygium* | *malaccense* |
| Myrtaceae | *Syzygium* | *nemorale* |
| Myrtaceae | *Syzygium* | *tierneyanum* |
| Lamiaceae | *Tectona* | *grandis* |
| Combretaceae | *Terminalia* | *calamansanai* |
| Combretaceae | *Terminalia* | *catappa* |
| Combretaceae | *Terminalia* | *whitmorei* |
| Vitaceae | *Tetrastigma* | *lauterbachianum* |
| Malvaceae | *Thespesia* | *populnea* |
| Rubiaceae | *Timonius* | *longitubus* |
| Rubiaceae | *Timonius* | *timon* |
| Boraginaceae | *Tournefortia* | *argentea* |
| Ulmaceae | *Trema* | *orientalis* |
| Ulmaceae | *Trema* | *tomentosa* |
| Poaceae | *Tripsacum* | *andersonii* |
| Poaceae | *Tripsacum* | *laxum* |
| Apocynaceae | *Tylophora* | sp. |
| Rubiaceae | *Uncaria* | *lanosa* |
| Malvaceae | *Urena* | *lobata* |
| Annonaceae | *Uvaria* | *macrophylla* |
| Fabaceae | *Vigna* | *marina* |
| Lamiaceae | *Vitex* | *cofassus* |
| Lamiaceae | *Vitex* | *trifolia* |
| Asteraceae | *Wollastonia* | *biflora* |
| Polygalaceae | *Xanthophyllum* | *papuanum* |
| Araceae | *Xanthosoma* | *sagittifolium* |
| Meliaceae | *Xylocarpus* | *granatum* |
| Zingiberaceae | *Zingiber* | *officinale* |
| Zingiberaceae | *Zingiber* | *zerumbet* |
