# Supplementary material for: Prestige and homophily predict network structure for social learning of medicinal plant knowledge
Source: PLoS One. 2020 Oct 8;15(10):e0239345. doi: 10.1371/journal.pone.0239345 (PMC7544085; doi:10.1371/journal.pone.0239345)
Supplement: S2 Appendix — (DOCX) [file pone.0239345.s021.docx]

| Category # | Category | Symptoms within category |
| --- | --- | --- |
| 1 | A General and unspecified | General health, malaria, fever/hot body, tuberculosis, measles, chicken pox, “any sickness”, leprosy, yellow fever, dengue fever, rubella, “swine flu” |
| 2 | B Blood, blood forming organs, lymphatics, spleen | Spleen illness, swollen lymph gland in armpit, swollen lymph nodes in neck |
| 3 | D Digestive | Diarrhea, dysentery, toothache, ulcer, rectal prolapse, liver problem, to eat well/hookworm, mumps, appendix problem, vomiting, constipation, dry mouth, bone stuck in throat |
| 4 | F Eye | Conjunctivitis, ants in eye, eye pain |
| 5 | H Ear | ear infection |
| 6 | K Circulatory | Low blood pressure, stroke, high blood pressure, edema |
| 7 | L Musculoskeletal | body pain, arthritis, bruises, break/sprain |
| 8 | N Neurological | Headache, dizziness, paralysis, epilepsy |
| 9 | P Psychological | Restless child, lunacy, bedwetting, spiritual attacks, protection of garden/house/property, enchantment to fight/play/remember/run/speak/sleep, love enchantment, divination, tame animals, charm, change weather |
| 10 | R Respiratory | cough/flu, pneumonia/cold, asthma, nasal congestion, sore throat |
| 11 | S Skin | *Tinea imbricata*, centipede bite, snake bite, cuts, burns, sores, lice, itchy skin, boils, yaws, hair problems, fish sting, itching from plants, peeling skin, pimples, nail problems, carbuncle, abscess, plantar warts |
| 12 | T Endocrine, metabolic and nutritional | Diabetes, "cancer" |
| 13 | U Urology | Urinary tract infection, kidney problem |
| 14 | W Pregnancy, childbirth, family planning | Abortion, childbirth, birth control, postpartum preeclampsia, infertility, choose sex of baby |
| 15 | X Female genital system and breast | gonorrhea/syphilis, excess menstrual bleeding, lactation aid, “AIDS”, breast cancer |
| 16 | Y Male genital system | gonorrhea/syphilis, hernia, penis enlargement, “AIDS” |
| 17 | Medicines to treat animals | Pig, dog, chicken, cat |

**S2 Appendix. List of Illnesses and Illness Categories (based on International Classification of Primary Care).**
